# Mechanisms of Tumor-Lymphatic Interactions in Invasive Breast and Prostate Carcinoma

**DOI:** 10.3390/ijms21020602

**Published:** 2020-01-17

**Authors:** Leticia Oliveira-Ferrer, Karin Milde-Langosch, Kathrin Eylmann, Maila Rossberg, Volkmar Müller, Barbara Schmalfeldt, Isabell Witzel, Jasmin Wellbrock, Walter Fiedler

**Affiliations:** 1 Department of Gynecology, University Medical Center Hamburg-Eppendorf, Martinistr. 52, 20246 Hamburg, Germany; milde-langosch@gmx.de (K.M.-L.); k.eylmann@uke.de (K.E.); m.rossberg@uke.de (M.R.); v.mueller@uke.de (V.M.); b.schmalfeldt@uke.de (B.S.); i.witzel@uke.de (I.W.); 2 Department of Hematology and Oncology, University Medical Center Hamburg-Eppendorf, Martinistr. 52, 20246 Hamburg, Germany; j.wellbrock@uke.de (J.W.); fiedler@uke.de (W.F.)

**Keywords:** lymphatic endothelial cells, lymphatic metastasis, co-culture, breast cancer, prostate cancer

## Abstract

During the last few years, diverse studies have shown that tumors can actively interact with the lymphatic system and promote metastases development. In order to examine the molecular mechanisms involved in this interaction, we co-cultured tumor and lymphatic endothelial cells (LEC) and subsequently analyzed the molecular alterations of LECs. Therefore, LECs were co-cultivated with either a highly or weakly metastatic breast cancer cell line using contact (mixture) and non-contact (transwell) co-cultures. mRNA profiles from LECs were subsequently analyzed for genes specifically induced by highly metastatic tumor cells (“metastatic specific”). Among the up-regulated “metastatic specific” genes, we found candidates involved in cell cycle, cell adhesion and motility (BST2, E-selectin, and HMMR), cytokines (CCL7, CXCL6, CXCL1, and CSF2) and factors of the complement system (C1R, C3, and CFB). Among the down-regulated genes, we detected the hyaluronan receptor STAB2, angiogenic factor apelin receptor (APLNR), and the glycosylation enzyme MAN1A1. In an additional prostate cancer co-culture model, we could confirm a “metastatic specific” upregulation of E-selectin and CCL7 in LECs after interaction with the prostate cancer cell lines LNCAP (highly metastatic) and DU145 (weakly metastatic). These data allowed us to identify a set of genes regulated in LECs during in vitro communication with cancer cells, which might subsequently facilitate lymphatic metastasis.

## 1. Introduction

Metastatic spread of tumor cells to distant organs is the main cause of mortality of cancer patients. Here, tumor cell intravasation into blood or lymph vessels represents a key step during the metastatic process. While hematogenous metastasis has been extensively investigated, the mechanisms by which lymphatics contribute to tumor cell dissemination remain relatively ignored. Over decades, metastasis through the lymphatic route was referred to as a passive process mainly caused by the natural drainage of detached tumor cells. However, intensive research during the last few years has shown that tumor cells and lymphatics actively interact, leading to lymphatic vessel growth (lymphangiogenesis). This interaction still promotes the access and subsequent transport of tumor cells through the lymphatic vessels to the proximal lymph nodes [1].

Currently, it is well accepted that lymphatic vessels are the main dissemination route to the lymph nodes, whereas their contribution to distant metastasis formation is still controversial. Lymphatic involvement is certainly an indicator of aggressiveness in many human cancers and lymph node status represents one of the most important parameters for patient prognosis and therapeutic strategies. 

Upon identification of specific markers for histological identification of lymphatic vessels in tissue, extensive research on tumor-induced lymphangiogenesis has been done. During the 1990s, the main growth factors regulating growth of lymphatic vessels, namely vascular endothelial growth factor (VEGF)-C and VEGF-D and their cognate receptor VEGR-3, were identified [2,3,4], and since then, many preclinical studies have shown the essential role of this signaling axis for tumor growth and metastatic spread [5,6,7,8,9]. A negative correlation between VEGF-C expression and lymphangiogenesis with patient outcomes could also be corroborated in diverse clinical studies [10,11,12,13]. Thus, tumor-induced lymphangiogenesis is thought to promote metastasis by increasing the probability of cancer cells entering into the newly generated lymphatic capillaries. On the other hand, recent studies have suggested that lymphatic endothelial cells are also able to communicate and affect tumor cells by secretion of growth factors, cytokines, and chemokines but also by upregulating certain adhesion molecules. Chemokine (C-C Motif) Ligand 21 (CCL21), a chemokine basically expressed by lymphatic endothelial cells [14,15], has been suggested to attract melanoma and breast cancer cells expressing its receptor Chemokine (C-C Motif) Receptor 7 (CCR7) [16,17]. Actually, an association between CCR7 expression in tumor cells and lymph node involvement has been confirmed in several clinical studies [18,19]. Moreover, a strong Chemokine (C-X-C Motif) Ligand 12 (CXCL12) expression is frequently present in lymph nodes, liver, lung, and bone marrow, which are favored metastatic sites of breast cancer cells expressing the respective receptor Chemokine (C-X-C Motif) Receptor 4 (CXCR4) [17]. In vivo studies have further shown that inhibition of this signaling axis reduces the ability of tumor cells to disseminate, particularly the lung and the lymph nodes. The macrophage mannose receptor I (MR) and Stabilin-1 (STAB1 or CLEVER-1) have been suggested to mediate cancer cell adhesion to lymphatic endothelium [20,21] and their expression on tumor lymphatic vessels has been associated with increased lymph node metastases [22]. In this context, the MR has been found to be a ligand for L-selectin, which is expressed in lymphocytes but also in cancer cells and mediates binding to the lymphatic endothelium. Here, L-selectin overexpressing tumor cells tended to metastasize to both the draining lymph and peripheral nodes, whereas cancer cells lacking L-selectin expression did not [23]. Moreover, certain adhesion molecules previously described in the context of immune cell traffic have been shown to be also involved in the intra- and extravasation process of tumor cells from the lympho-vascular compartment [24].

Regarding the different metastatic potential of cancer cells in vivo, we hypothesize that tumor cells might differ in their interaction with LECs. Thus, the aim of our study is to investigate whether metastatic tumor cells are able to induce specific molecular changes in lymphatic endothelium, which in turn might promote metastatic spread by affecting, i.e., adhesion, intravasation, proliferation, or migration of tumor cells. For this purpose, we used in vitro co-culture systems containing metastatic tumor cells and lymphatic endothelial cells (LEC) with contact to each other (mixture/contacting) as well as without direct contact (transwell/non-contacting) and subsequently analyzed the molecular alterations underwent by lymphatic cells by microarray analysis.

## 2. Results

### 2.1. Co-Cultures of Lymphatic Endothelial Cells (LEC) with Breast Cancer Cell Lines MCF7 and MDA-MB231

**Figure 1:**
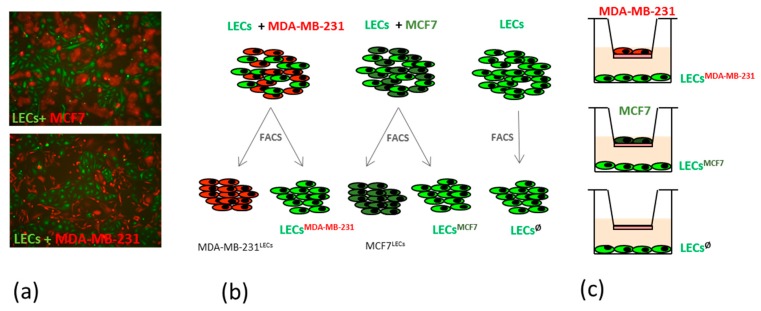
Principle of co-culture experiments. (**a**) Human dermal lymphatic endothelial cells (Lymphatic Endothelial Cells (LECs); green) and breast cancer cell lines MCF7 or MDA-MB231 (red) were co-cultured in a 1:1 ratio for 48 h and the morphology and association pattern was observed; (**b**) Schematic representation of mixed co-culture systems and subsequent cell sorting for further molecular characterization; (**c**) Schematic representation of no-contact co-cultures using a transwell system.

In order to analyze the interactions between metastatic breast cancer cells and lymphatics, we co-cultivated primary lymphatic endothelial cells (LECs) with the highly metastatic cell line MDA-MB231 and analyzed the phenotypic changes in LECs in comparison with those LECs of co-cultures containing the low metastatic MCF7 cells or with the LECs in mono-cultures. Endothelial cells were previously tracked with the green fluorescent dye CMFA and breast cancer cell lines stably transduced with a lentiviral vector containing a red fluorescent dye (Figure 1a). Using fluorescence microscopy, we observed co-cultures with either 1:1, 1:2, or 1:3 ratios (LEC:cell line) during a period of up to 5 days. Here, we did not observe differences in cell association patterns or in the morphology of endothelial cells co-cultivated with weakly vs. highly metastatic breast cancer cells.

### 2.2. Comparative Gene Expression Profile in LECs after Contacting Co-Culture with a High vs. a Low Metastatic Breast Cancer Cell Line

We were further interested in the molecular changes that LECs might undergo after direct interaction with metastatic cells and, therefore, cell sorting and subsequent RNA isolation of both cell populations were performed after 48 h co-cultivation of LECs with MDA-MB231 as well as with MCF7 cells or LECs alone (Figure 1b).

cDNA arrays with mRNA isolated from LECs co-cultivated with the highly metastatic cell line MB-MDA231 (LEC^MDA-MB231^), LECs co-cultivated with the low metastatic cell line MCF7 (LEC^MCF7^), or LECs in mono-culture (LEC^Ø^) were performed in triplicate. Since we were mainly interested in gene deregulation induced by metastatic cancer cells, we first analyzed those genes with significantly increased or decreased expression of at least twofold in LEC^MDA-MB231^ compared with LEC^MCF7^. Statistical analyses were performed to identify the 200 top deregulated genes, from which 139 were induced and 61 were repressed.

We further examined whether these 200 genes were only deregulated in LECs after co-cultivation with highly metastatic cells (MDA-MB231) or also in those LECs co-cultured with the low metastatic cell line (MCF7). Those genes showing a significant de-regulation (fold change >2) in LEC^MCF7^ versus LEC^Ø^ were defined as “no-metastatic specific” and excluded from subsequent analyses (Table S1). Following these criteria, we established a list of genes that were exclusively deregulated in LECs by highly metastatic breast cancer cells (Table S2). Among these “metastatic specific” upregulated genes, we found candidates involved in cell cycle and cytoskeleton (i.e., CDC6, claspin, and diverse kinesin family members), cell adhesion and motility (i.e., BST2, E-selectin, and HMMR), but also cytokines (i.e., CCL7, CXCL6, CXCL1, and CSF2) and factors of the complement system (i.e., C1R, C3, and CFB). Among the downregulated genes, we detected the hyaluronan receptor STAB2, angiogenic factor apelin receptor (APLNR) or glycosylation enzymes such as the mannosidase MAN1A1, and the acetylglucosaminyltransferase MGAT4A.

Validation at the mRNA level was performed using qRT-PCR for a representative group of genes and corroborates upregulation of all analyzed factors (Table 1). Fold change values achieved with qRT-PCR were generally higher than those from microarrays, confirming the fact that standard thresholds are often too stringent in microarray analysis [25].

**Figure 2:**
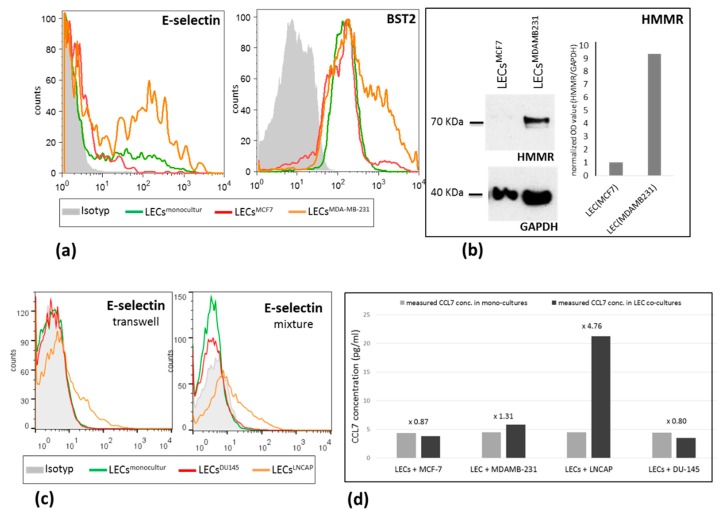
Protein expression level of E-selectin, BST2, and HMMR in LECs after different culture conditions. (**a**) E-selectin and BST2 expression, measured by flow cytometry, is strongly increased in LECs after co-culture (mixture) with MDA-MB231 cells in comparison to LECs co-cultured with MCF7 cells or LECs in mono-culture; (**b**) Increased expression of HMMR was detected using Western blot in LECs^MB-MDA231^ versus LECs^MCF7^; (**c**) Increased E-selectin expression was measured by flow cytometry in LECs after co-culture (mixture as well as transwell) with LNCAP cells in comparison to LECs co-cultured with DU145 cells or LECs in mono-culture; (**d**) CCL7 levels were measured by ELISA in the supernatant of mono- and co-culture systems. Increased amount of CCL7 was detected in co-cultures of LECs/MDA-MB231 and LECs/LNCAP in comparison to monocultures or the LECs/MCF7 and LECs/DU145 systems. BST2 and E-selectin were analyzed using flow cytometry directly on co-cultures without previous cell sorting by double staining with cell tracker. For HMMR, Western blot analyses were performed with co-cultured LECs after cell sorting, as previously described.

For non-secreted factors, namely BST2, E-selectin, and HMMR, enhanced expression in LECMDA-MB231 could be additionally corroborated at the protein level. BST2 and E-selectin were analyzed using flow cytometry directly on co-cultures without previous cell sorting by double staining with cell tracker. Here, BST2 and E-selectin were more highly expressed within the endothelial subpopulation of LECs+MDA-MB231 co-cultures compared with those from the LECs+MCF7 mixture (Figure 2a). For HMMR, Western blot analyses were performed with co-cultured LECs after cell sorting, as previously described. HMMR was expressed on LEC^MDA-MB231^, whereas it could not be detected in LEC^MCF7^ (Figure 2b).

### 2.3. LEC Response after Transwell Co-Culture with a High vs. Low Metastatic Breast Cancer Cell Line

In order to clarify whether gene deregulation in LECs was induced through soluble factors secreted by the breast cancer cell line (paracrine) or was rather induced after cell–cell interaction (juxtacrine), we additionally performed no-contacting co-culture experiments using transwell inserts. LEC response after paracrine culture with MDA-MB231 and MCF7 cells was measured by qRT-PCR. Similar to contact cultures, we found an up-regulation of most cytokines (except CCL7) and all factors of the complement system in LEC^MDA-MB231^ compared with LEC^MCF7^, whereas no deregulation of the adhesion and motility factors (E-selectin, BST2, or HMMR) could be observed (Table 1).

### 2.4. Gene Expression Analysis in LECs after Co-Culture with a Highly vs. a Non-Metastatic Prostate Cancer Cell Line

We were further interested in whether gene deregulation observed in LECs co-cultured with metastatic breast cancer cells was also relevant in a second cancer model with strong lymphatic involvement. To this aim, we chose prostate cancer since this tumor entity frequently shows lymphatic metastasis. In this context, two prostate cancer cell lines (LNCAP and DU145) with different metastatic behavior were used in additional co-culture experiments. For LNCAP, a high metastatic potential has been described, whereas DU145 cells are known as poorly lymph node metastatic [26]. As previously described for breast cancer cell lines and LECs, contacting (mixture) and non-contacting (transwell) co-cultures with lymphatic endothelial cells, tracked with the green fluorescent dye CMFA, and DU145 or LNCAP, stably transduced with a lentiviral vector containing a red fluorescent dye, were performed.

In a first approach, we analyzed mRNA expression in LECs^DU145^ and LECs^LNCAP^ after mixture and transwell co-culture for 48 h. We focused on those genes represented in Table 2 that were significantly deregulated and validated in the breast cancer/LEC model. Here, we found that the majority of the analyzed genes were more highly up-regulated in LECs induced by LNCAP than in those co-cultured with DU 145 cells (Table 2). Remarkably, the mRNA levels of E-selectin and CCL7 in LECs were not significantly affected by the direct interaction with the low-metastatic cell line DU145, whereas a huge increase was measured after co-cultivation with the high-metastatic LNCAP cells (fold change 37 and >100, respectively). In the transwell assay, we also observed an upregulation of both factors; however, it was in a less extensive manner (fold change 8.7 and 4.7, respectively). BST2 and CFB represent an exception, showing a 38- and 13-fold higher expression, respectively, in LECs^DU145^ vs. LECs^LNCAP^ after contacting co-culture. In line with the results from the breast cancer model, BST2 was not significantly deregulated in the transwell experiment, whereas the complement factor B (CFB) was 6-fold overexpressed under these conditions. At the protein level, the upregulation of E-selectin in LECs co-cultured with LNCAP cells (LECs^LNCAP^) could be validated using FACS in both transwell and mixture models (Figure 2C). As expected, co-cultivation with the less metastatic cell line DU145 did not lead to the deregulation of this factor in the endothelial cells. Additionally, CCL7 levels were measured by ELISA in the media of mono-cultured LECs, MCF7, MDA-MB231, DU145, and LNCAP cells as well in the supernatant of mixed co-cultures containing LECs and each one of the mentioned cell lines. CCL7 was slightly reduced in the supernatant of LEC/MCF7 (0.87-fold) and LEC/DU145 co-cultures (0.8-fold) in comparison with the total amount calculated by adding CCL7 levels measured in each single culture (LEC+MCF7 and LEC+DU145). In contrast, co-cultivation of LECs with the highly metastatic cell lines MDA-MB231 or LNCAP led to increased CCL7 secretion of 1.3-fold and 4.7-fold, respectively. Based on the mRNA data, we assume that the enhanced protein expression and release of CCL7 during co-culture conditions is mainly mediated by the LECs. However, in this setting, we cannot totally exclude that the tumor cell lines, upon interaction with the LECs, also increase their CCL7 secretion.

## 3. Discussion

The active role of lymphatics during tumor progression and metastasis has been clearly documented during the last few years [1]. Nevertheless, little is known about the mechanisms involved in lymphatics activation and whether this process requires a direct interaction between metastatic tumor cells and lymphatic endothelium or is mainly driven by paracrine factors. In this study, we describe molecular alterations in lymphatic endothelial cells (LECs) after interaction with highly metastatic breast cancer or prostate cancer cells, but not after co-culture with weakly metastatic cell lines, by using two different co-culture systems in vitro. We have chosen breast and prostate cancer models since these are tumor entities with strong lymphatic involvement. We found commonly deregulated factors in LECs in both metastatic tumor cell models, namely a strong up-regulation of E-selectin, cytokine CCL7, and complement component 3 (C3). Further, we observed that deregulation of certain factors such as BST2, E-Selectin, HMMR, and CCL7 in LECs requires a direct interaction with the tumor cell, whereas higher expression levels of most cytokines (CXCL1, CXCL2, CXCL6, CSF2) and the complement system molecules (C1R, C3 and CFB) can also be induced in LECs by paracrine signaling.

Although the involvement of the lymphatic system has been traditionally accepted as a marker of aggressiveness and a potent predictor of patient survival, for decades, lymphatic vessels have been considered to be only passively involved in tumor spread. This view has drastically changed upon identification of VEGF-C and VEGF-D as lymphangiogenic factors [2,3] and the subsequent implication of these factors and their cognate receptor VEGFR-3 in lymph node and distant metastasis [27,28,29]. Besides the identification of further pro-lymphangiogenic factors such as PDGF-BB, IL7, FGF2, or ANG2 [30], diverse groups have described molecular changes in activated lymphatics versus quiescent vessels. This includes the deregulation of cytokines, chemokines, as well as cell-adhesion molecules and matrix proteins [31,32]. We were interested in the changes specifically induced in LECs upon stimulation by metastatic tumor cells, which might, in turn, facilitate tumor cell spread through lymphatics and further colonization of lymph nodes and distant organs. In this context, we analyzed molecular alterations in LECs caused by both a non-metastatic and a highly metastatic breast cancer cell line after direct co-cultivation. Here, we found various commonly deregulated factors in both cell lines such as the chemokines CXCL2 and CCL5 and the cytokines IL6 and IL8 (Table S2) that were not taken into consideration for further analysis. In contrast, factors upregulated in LECs only after co-cultivation with the metastatic cell line were considered as “metastatic specific”. Among these genes, we identified factors related to cell cycle, cell motility, adhesion, cytoskeleton, cytokines, chemokines, complement system, or protein glycosylation. Deregulation of these genes induced by direct contact with the highly metastatic cell line MDA-MB231 could be further validated by qRT-PC, WB, and/or FACS for an exemplary group of genes, including soluble factors as well as membrane-bound proteins.

The highest upregulation in LECs after direct interaction with the tumor cells was observed for the cytokines CCL7 and CXCL6. Interestingly, an increased level of CXCL6 was also detected in the transwell assay, suggesting paracrine stimulation, whereas for CCL7, no significant up-regulation under these conditions was noticed. The association of these two factors with cancer metastasis could be corroborated in the LEC-prostate cancer model, where we found a strong up-regulation of CCL7 and CXCL6 in LECs after interaction with the metastatic cell line LNCAP, but not after co-culture with the non-metastatic cell line DU145. CCL7 has been described to activate immune cells via binding to the receptors CCR1, CCR2 and CCR3 [33]. Both tumor cell lines (MDAMB231 and DU145) have been previously shown to endogenously express CCL7 receptors [34,35]. Besides, direct interaction with tumor cells has been shown in different entities. Here, the CCL7/CCR3 signaling axis has been described to promote colon cancer metastasis in a ERK-JNK-dependent manner. In prostate cancer, CCL7, which is secreted by adipocytes, stimulates migration of CCR3-positive tumor cells and acts as a driving force for cancer progression in obesity [36,37]. A similar scenario might take place in breast cancer since the CCL7 receptor CCR3 has been described to be widely expressed in breast tumor cells [38]. Our results suggest that metastatic tumor cells induce CCL7 expression in LECs that, in turn, might attract further CCR3-positive tumor cells promoting tumor cell dissemination through lymphatics. Lee et al. have described an analogous mechanism based on the tumor cell-induced activation of lymphatics in pre-metastatic sites. Here, upregulation of CCL5 in lymphatic endothelial cells promotes further tumor cell dissemination [39]. We also found an up-regulation of CCL5 after interaction with the metastatic cell line MDA-MB231. Nevertheless, since a significant upregulation was also found with the less metastatic cell line MCF7, CCL5 was not regarded as “metastasis-specific”.

CXCL6 is principally associated with neutrophil chemotaxis, thereby regulating angiogenesis and immunosuppression that, in turn, promote tumor progression [40,41]. Further, CXCL6 acts as an autocrine growth factor in tumor cells itself, i.e., in small cell lung cancer cells expressing its cognate receptors (CXCR1 and CXCR2) [42]. In this context, the treatment with CXCL6-neutralizing antibodies led to a reduced tumor cell growth and lymphatic metastasis in a melanoma mouse model [43]. In line with our data, CXCL6 and CCL7 were previously found to be upregulated in tongue cancer cell-induced LECs [32], suggesting that these factors represent a common mechanism of lymphatics-induced tumor cell progression.

Interestingly, three components of the complement system, namely C3, C1R, and CFB, also showed a significant upregulation after interaction with the metastatic cell line MDA-MB231 and two of them (C3 and C1R) could be validated in the prostate cancer model. In addition to its role in innate immunity and its involvement in the adaptive immune response, the end products of complement activation, so-called anaphylatoxins, and their receptors promote cell dedifferentiation, proliferation, and migration as well as apoptosis inhibition. High expression of complement and complement regulatory proteins has also been shown in cancer. Cho et al. described an association between C3 and C5aR mRNA levels with decreased overall survival in patients with ovarian and lung cancer. Here, cancer cells secreted complement protein themselves and stimulated tumor growth upon autocrine activation. In the same study, C3 deficiency additionally showed an effect on immune cell recruitment [44].

Three membrane-associated proteins, namely E-selectin, BST2, and HMMR were found to be upregulated in LECs after direct interaction with highly metastatic tumor cells. However, none of them was affected in the transwell assay, suggesting that cell–cell contact and juxtacrine signaling might be required for this activation. The induction of E-selectin could be further validated in the prostate-LEC model. The glycoprotein E-selectin is known to facilitate the attachment or tethering of flowing leukocytes to vascular endothelial cells (BECs) in the vessel wall via labile binding to sialylated carbohydrates. For tumor cells, a similar mechanism has been described in the context of the hematogenous metastasis [45]. Although the endogenous E-selectin expression level in LECs is considerably lower than in BECs [46], some groups have reported on immune cell adhesion to activated LECs via E-selectin [47,48]. To our knowledge, this is the first report on tumor-induced E-selectin activation in LECs in the context of lymphatic metastasis and it suggests that tumor cell adhesion to lymphatics might also be enhanced via E-selectin up-regulation.

Similarly, E-selectin has been previously described to be up-regulated in vascular endothelial cells by the triple-negative cell line MDAMB123 [49,50]. However, other adhesion molecules, such as PECAM-1 and VE-Cadherin, that were shown to be downregulated in vascular endothelial cells after co-culture with MDMB231 cells, were not affected in LECs in our experiments [51]. To our knowledge, there is also no report of CCL7 induction on vascular endothelial cells after co-culture with MDAMB123 cell or with other triple-negative breast cancer cell lines. These facts suggest that the mechanisms of vascular and lymphatic endothelial cell activation by metastatic breast cancer cells might rather differ.

The bone marrow stromal antigen 2 (BST2)/tetherin has been originally reported to be involved in the reduced release of the human immunodeficiency virus from infected cells [52]. Recently, a tumorigenic role of BST2 has been suggested although the mechanism has not been completely clarified. In breast cancer cells, BST-2 mediates cancer cell adhesion and anchorage-independent growth thereby promoting tumor cell survival through inhibition of anoikis [53]. In vascular endothelial cells, IFNγ-induced up-regulation of BST2 mediates adhesion to monocytes [54]. Whether BST2, similarly to E-selectin, might facilitate adhesion not only to monocytes but also to tumor cells has to be clarified in further studies. Similarly, HMMR, the receptor for HA-mediated motility, plays a key role in the formation of new blood vessels [55], whereas its function in lymphatics has not yet been investigated.

One limitation of the co-culture models in this study is the lack of other cell types, which are naturally present in the tumor microenvironment and might also contribute to the activation of lymphatics. In this context, we cannot conclude from our in vitro study that the direct interaction between metastatic tumor cells and lymphatics and the subsequent activation of LECs is “the unique mechanism” of lymph node metastasis. Yamaguchi et al. showed that adipose tissue invasion at the tumor periphery has a significant impact on lymph node metastasis and, in turn, on the prognosis of patients with invasive breast cancer [56]. Further, a key role of tumor-educated B cells on breast cancer lymph node metastasis has been recently described. Here, B cell-derived pathogenic antibodies target the tumor antigen HSPA4 and promote the formation of the premetastatic niche in tumor-draining lymph nodes [57]. Further, we are aware that MCF7 and MDAMB123 cells are biologically different cell types. Consequently, not all changes observed in LECs after co-culture experiments might be only the result of their different metastatic potential. Therefore the importance of analyzing the second prostate cancer model of lymphatic metastasis. Here, several factors could not be validated, but E-selectin and CCL7 were confirmed to be involved in lymphatic activation.

Regarding the signaling mode of lymphatics activation, we obtained contradictory results. In the breast cancer model, we found upregulation of the membrane-associated factors, E-selectin, BST2, and HMMR, as well as the cytokine CCL7 only under mixture-culture conditions. This suggests that direct interaction of both cell types, a juxtacrine signaling, is necessary for their stimulation. In the prostate model, we could not validate this finding. Here, E-selectin and CCL7 were also up-regulated in the transwell-assay indicating paracrine stimulation. Overall, cytokines (excepting CCL7 in the breast cancer and CFB in the prostate cancer system, respectively) are upregulated under both transwell and mixture conditions. Here, we assume that physical contact between tumor and lymphatic endothelial cells is not imperative in order to activate LECs. Nevertheless, the extent of the upregulation is much higher under contacting co-cultures, which would imply that LEC activation is more pronounced when both cell populations have physical contact. Our hypothesis is that tumor cells activate LECs in situ, creating a pre-metastatic niche that leads to further recruitment of more distant tumor cells, thereby increasing the extent of invasion and, in turn, of lymphatic metastasis.

## 4. Materials and Methods

### 4.1. Cell Culture and Co-Culture Systems

Human mammary cancer cell lines MDA-MB231 and MCF7 were cultivated, as described before (Bamberger et al. (2001); Milde-Langosch et al. (2001, 2004)). Human prostate cancer cell lines DU 145 and LNCaP were cultivated in RPMI (Gibco, Grand Island, NY, USA) supplemented with 10% fetal bovine serum. For contacting co-cultures, mammary and prostate cell lines expressing the fluorescent protein mCherry were generated. Here, co-transfection of the LeGO-iC2-Puro+-Luc2 vector and the packaging plasmids phCMV-VSV-G, pMDLg/pRRE and pRSV-Rev was performed in HEK293. After 24 h, lentiviral supernatants were collected and lentiviral transductions were performed on MDA-MB231, MCF7, DU 145, and LNCaP cells that were previously seeded in 24 well-plates. Transduced cell lines were treated after 48 h with 2 µg/mL puromycin for selection. Human dermal lymphatic endothelial cells (LECs; HDLECs, Promocell, Heidelberg, Germany) were cultured in endothelial cell growth medium MV2 (PromoCell), containing with endothelial cell growth medium MV2 SupplementMix (PromoCell). Endothelial cells were used between passages 3 to 5. Where specified, LECs were stained with the fluorescent CellTracker Green CMFDA dye (Invitrogen, Thermo Fisher Scientific, Waltham, MA, USA), as described by the supplier.

For contacting co-cultures, a cancer cell line expressing mCherry (MDA-MB231 or MCF7 or DU 145 or LNCaP) and LECs tracked with green fluorescent dye were plated at 80–90% confluency at a 1:1 ratio in basal medium MV2 (Promocell) containing 2% FCS and cultured for 48 h. Control cultures containing one cell type were grown under identical conditions. Co-cultured and control cells were collected using Accumax (eBioscience, San Diego, CA, USA) and resuspended in PBS buffer containing 0.5% EDTA.

For non-contacting co-cultures (transwell), LECs were seeded on the lower surface of a 6-well plate Transwell system at 90% confluency and one cancer cell line (MDA-MB231 or MCF7 or DU 145 or LNCaP) was plated in the insert (upper surface) at 70% confluency. The co-culture was incubated in basal medium MV2 (Promocell) containing 2% FCS for 48 h and cells were separately collected using accumax and subsequently processed for RNA isolation.

### 4.2. Cell Sorting

Prior to gene expression analysis or protein isolation, cells in contacting co-cultures were separated by fluorescence activated cell sorting (FACS). Here, 1 × 10^6^ tumor cells mCherry (MDA-MB231 or MCF7 or DU 145, or LNCaP) and 1 × 10^6^ LECs tracked with green fluorescent dye were plated in basal medium MV2 (Promocell) containing 2% FCS and cultured for 48 h. Control cultures containing one cell type (2 × 10^6^ cells) were grown under identical conditions. Subsequently, co-cultured and control cells were collected using Accumax (eBioscience, San Diego, CA, USA) and resuspended in PBS buffer containing 0.5% EDTA. Resuspended cells were filtered through a 30 μm cell strainer, and cell sorting was performed in a FACSAria III (BD Biosciences, San Jose, CA, USA). Sorting gates were set by the fluorescence intensity of CMFDA dye and mCherry. At least 300,000 events per sorted cell type were collected in cell type-specific medium containing 10% FCS. Subsequently, cells were spun down and mRNA or proteins were extracted for further analysis

### 4.3. RNA Isolation and PCR

LECs pellets from contacting co-cultures were achieved after sorting as described above and resuspended in RLT buffer solution containing β-mercaptoethanol (Qiagen, Hilden, Germany). LECs from transwell co-cultures were directly resuspended in RLT buffer solution containing β-mercaptoethanol (Qiagen). RNA extraction was performed from RLT lysates using an RNeasy kit (Qiagen, Hilden, Germany), as described in the manufacturer’s instructions. RNA concentration and quality were determined photometrically using Nanodrop (Peqlab, VWR International GmbH, Erlangen, Germany). cDNA synthesis (500 ng RNA) was performed using the Transcriptor First Strand Synthese kit (Roche) and subsequent quantitative RT-PCR experiments were conducted using a capillary-based Light Cycler (Roche, Basel, Switzerland) and the SYBR Premix Ex Taq (Takara). Primer sequences are listed in Supplementary Table S3. Samples were analyzed in duplicate and averaged and data were analyzed based on the ΔΔ*C*_t_ method.

### 4.4. Microarray Analysis

RNA quality controls from LEC cells as well as subsequent microarray analysis and data evaluation were performed by ATLAS Biolabs GmbH (Berlin, Germany). Briefly, cDNA synthesis and labeling, using 500 ng total RNA as starting material, were performed following Ambion^®^ WT Expression kit instructions (Applied Biosystems for Affymetrix^®^ GeneChip^®^ Whole Transcript (WT) Expression Arrays, Foster City, CA, USA). For these experiments we used an Affymetrix Human Gene 1.0 ST array (Affymetrix Inc., Santa Clara, CA, USA). Annotation data have been jointly pre-processed using the robust multi-array average (RMA) before applying statistical analysis.

### 4.5. Western Blot

Protein extraction and Western blot analysis were performed as described [58] with following antibodies: rabbit polyclonal anti HMMR (1:100; Origene, Rockville, USA) and GAPDH monoclonal antibody (1:1000; Santa Cruz, Dallas, TX, USA).

### 4.6. Enzyme-Linked Immunosorbent Assay for Quantitative Detection of Human CCL7 (MCP-3)

Cell supernatants from co-culture systems (LECMCF7, LECMDA-MB231, LECLNCAP, LECDU 145) and from each single cell line were collected and CCL7 content were measured by ELISA (Invitrogen, Cat No 88-50700, Carlsbad, CA, USA), according to the manufacturers protocol. Each sample was analyzed in triplicates in three independent experiments.

### 4.7. Flow Cytometry

For assessment of BST2 and E-selectin in LECs after contacting co-culture with a carcinoma cell line, co-cultured cells—containing LECs, previously tracked with green fluorescent dye, and a carcinoma cell line expressing the fluorescent dye mCherry (MDA-MB231 or MCF7 or DU 145, or LNCaP)—were collected after 48 h incubation using Accumax, washed with PBS, and resuspended in PBS with 1% BSA. Subsequently, cells were stained with the anti-BST2 antibody (BioLegend, San Diego, CA, USA) or the anti-E-selectin-antibody (BD Bioscience Franklin Lakes, New Jersey, NJ, USA) as well as with the corresponding isotype controls. The mixed cell population was gated based on the green fluorescent dye CMFDA, corresponding to the LECs, and fluorescence intensity of BST2 or E-selectin was evaluated within this cell population (LEC^MCF7^, LEC^MDA-MB231^, LEC^LNCAP^, LEC^DU145^) related to the isotype staining.

### 4.8. Statistic

In order to identify induced and repressed genes between the two experimental groups based on the microarray data, a *t*-test was performed using bioconductor package limma. A list with the top induced and repressed genes was supplied by ATLAS Biolabs GmbH (Berlin, Germany).

## 5. Conclusions

In summary, our results show that highly metastatic tumor cells specifically activate lymphatic endothelial cells that might further promote metastasis. We were able to identify two factors, namely E-selectin and the chemokine CCL7, which were upregulated in lymphatics in both breast and prostate cancer, representing two different cancer models with strong lymphatic metastatic involvement.

## Figures and Tables

**Table 1:** Validation of selected “metastatic specific” genes, deregulated in LECs^MDA-MB-231^ versus LECs^MCF7^ after 48 h.

		LECs Co-Cultivated with MDA-MB-231 vs. MCF7			
Gene Symbol	Gene Description	MIXTURE			TRANSWELL
		Fold Change			Fold Change
Adhesion and Motility		MICROARRAY	Validation qRT-PCR	Validation WB/FACS	qRT-PCR
BST2	bone marrow stromal cell antigen 2	2.86	7.33	FACS	−1.05
SELE	selectin E	4.10	9.58	FACS	−1.43
HMMR	hyaluronan-mediated motility receptor (RHAMM)	2.63	6.89	WB	−1.30
**Cytokines**					
CCL7	chemokine (C-C motif) ligand 7	21.50	127.56		−1.04
CXCL1	chemokine (C-X-C motif) ligand 1 (melanoma growth stimulating activity, alpha)	5.79	24.50		3.62
CXCL6	chemokine (C-X-C motif) ligand 6 (granulocyte chemotactic protein 2)	15.04	54.57		8.63
CSF2	colony stimulating factor 2 (granulocyte-macrophage)	3.55	177.29		3.25
**Complement System**					
C1R	complement component 1, r subcomponent	5.44	26.48		9.88
C3	complement component 3	11.82	46.50		7.57
CFB	complement factor B	6.22	36.55		10.79

LECs^MDA-MB-231^: LECs co-cultured with cell line MDA-MB-231; LECs^MCF7^: LECs co-cultures with cell line MCF7; WB = western blot; FACS = fluorescence activated cell sorting.

**Table 2:** Fold change expression of selected genes in LECs^LNCAP^ compared to LECs^DU145^ after 48 h.

	MIXTURE			TRANSWELL
Gene Symbol	LEC^LNCAP^ vs. LEC^DU145^	LEC^LNCAP^ vs. LEC^Ø^	LEC^DU145^ vs. LEC^Ø^	LEC^LNCAP^ vs. LEC^DU145^
SELE	36.89	53.82	1.46	8.72
BST2	−38.30	2.24	85.92	1.26
HMMR	1.14	−4.02	−4.60	−1.16
CCL7	>100	93.05	−1.80	4.76
CXCL6	>100	78.52	−14.30	74.29
CSF2	27.00	11.31	−2.40	13.50
C1R	4.16	98.02	23.59	5.11
CFB	−13.20	1.39	18.38	−6.15
C3	33.47	82.71	2.47	10.52

LECs^LNCAP^: LECs co-cultured with cell line LNCAP; LECs^DU145^: LECs co-cultures with cell line DU145; LECs^Ø^: LECs in monoculture.

## Supplementary Materials

The following are available online at https://www.mdpi.com/1422-0067/21/2/602/s1.

## Abbreviations

LEC	lymphatic endothelial cells
LEC^MDA-MB231^	lymphatic endothelial cells co-cultured with MDA-MB231 cells
LEC^MCF7^	lymphatic endothelial cells co-cultured with MCF7 cells
LEC^DU145^	lymphatic endothelial cells co-cultured with DU 145 cells
LEC^LNCAP^	lymphatic endothelial cells co-cultured with LNCAP cells
LEC∅	lymphatic endothelial cells in monoculture
FACS	fluorescence activated cell sorting
